# Computational Approaches to Prioritize Cancer Driver Missense Mutations

**DOI:** 10.3390/ijms19072113

**Published:** 2018-07-20

**Authors:** Feiyang Zhao, Lei Zheng, Alexander Goncearenco, Anna R. Panchenko, Minghui Li

**Affiliations:** 1 School of Biology and Basic Medical Sciences, Soochow University, Suzhou 215123, China; 1530416014@stu.suda.edu.cn (F.Z.); 1513401013@stu.suda.edu.cn (L.Z.); 2 National Center for Biotechnology Information, National Institutes of Health, Bethesda, MD 20894, USA; alexandr.goncearenco@nih.gov (A.G.); panch@ncbi.nlm.nih.gov (A.R.P.)

**Keywords:** cancer driver missense mutations, macromolecular stability, macromolecular interactions, conformational dynamics

## Abstract

Cancer is a complex disease that is driven by genetic alterations. There has been a rapid development of genome-wide techniques during the last decade along with a significant lowering of the cost of gene sequencing, which has generated widely available cancer genomic data. However, the interpretation of genomic data and the prediction of the association of genetic variations with cancer and disease phenotypes still requires significant improvement. Missense mutations, which can render proteins non-functional and provide a selective growth advantage to cancer cells, are frequently detected in cancer. Effects caused by missense mutations can be pinpointed by in silico modeling, which makes it more feasible to find a treatment and reverse the effect. Specific human phenotypes are largely determined by stability, activity, and interactions between proteins and other biomolecules that work together to execute specific cellular functions. Therefore, analysis of missense mutations’ effects on proteins and their complexes would provide important clues for identifying functionally important missense mutations, understanding the molecular mechanisms of cancer progression and facilitating treatment and prevention. Herein, we summarize the major computational approaches and tools that provide not only the classification of missense mutations as cancer drivers or passengers but also the molecular mechanisms induced by driver mutations. This review focuses on the discussion of annotation and prediction methods based on structural and biophysical data, analysis of somatic cancer missense mutations in 3D structures of proteins and their complexes, predictions of the effects of missense mutations on protein stability, protein-protein and protein-nucleic acid interactions, and assessment of conformational changes in protein conformations induced by mutations.

## 1. Introduction

Cancer is a complex disease that is driven by genetic alterations. Cancer genome sequencing projects have revealed vast numbers of somatic mutations [1,2], and the majority of these are expected to be passenger mutations (i.e., mutations having no direct or indirect effect on a selective growth advantage of tumor cells) [3]. A group of key mutations, called drivers, significantly alter normal cellular systems [4,5], providing a selective growth advantage to cancer cells [3] that becomes apparent during different stages of oncogenesis. A large number of mutations detected in cancer are single nucleotide variants (SNVs,), and those that alter amino acid sequences are called missense mutations. These mutations may affect protein structure/stability and disrupt protein interactions with other biomolecules, rendering proteins non-functional and potentially promoting tumor progression. Some missense mutations have been identified as drivers, such as the *BRAF* V600E mutation in melanoma [6] and the *KRAS* G12D and G12V mutations in colorectal cancer [7].

The key challenge in cancer research is to determine which mutations are likely to be drivers. Although mutations that are observed very frequently can be classified as drivers, many mutations discovered thus far are observed in a relatively small fraction of tumors [8]. Thus, methods that can identify driver or passenger mutations without explicitly relying on observed frequency counts are clearly needed [9]. Experimental methods, including functional studies in model organisms or in cultured cells using gene knockout or siRNA, are extremely useful for elucidating the function of individual mutated genes. However, they have limitations with respect to analyzing a large number of gene candidates from large-scale cancer genome projects. For missense mutations, one can considerably decrease the number of potential driver candidates by determining the functional impact of each mutation on proteins [10]. In addition, mutations that confer drug resistance should be identified. Overall, the binary driver-passenger model can and should be adjusted by taking into account additive pleiotropic effects of mutations [11,12]. Subcellular localization of proteins is also important to their biological functions and aberrant protein subcellular localization is closely correlated to cancer, such as primary human liver tumors [13] and breast cancer [14]. Knowing where a protein resides within a cell can give insight into identification of drug targets and drug design [15]. Several computational methods have been developed to determine the subcellular localization of proteins that deal with large-scale proteomic data [16,17].

**Figure 1:**
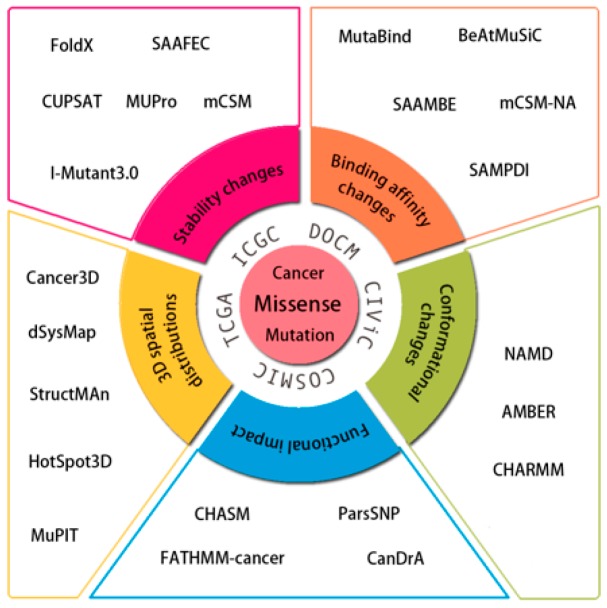
Overview of computational approaches and tools for identifying cancer driver missense mutations. Each method or tool was assigned to one of the five categories.

In this review, we focus on the description of computational approaches and tools to annotate cancer driver missense mutations. We divide the process of annotating functional and driver variants into five independent, but related, approaches (Figure 1). The first consists of analyzing the distribution of cancer somatic missense mutations in 3D structures of protein and protein complexes with protein-binding partners, nucleic acids and low molecular-weight ligands. These resources can help identify cancer drivers, drug biomarkers, or rationalize the mechanism of action. The second approach introduces computational methods for predicting the effects of missense mutations on protein stability, which may directly relate to their functional activity. Computational methods that accurately predict the effects of variations on protein stability may help identify functionally important mutations. The third group describes computational methods for predicting the effects of missense mutations on protein–protein and protein–nucleic acid interactions. A protein’s ability to establish highly selective interactions with macromolecular partners is a crucial prerequisite for proper biological function. A missense mutation affecting protein interactions may cause significant perturbations or complete abolition of protein function, potentially leading to disease. The fourth group introduces molecular dynamic simulations to assess changes in proteins and their conformations induced by mutations, which may aid in the detection of cancer drivers and elucidation of molecular mechanisms. The fifth approach discusses several statistical methods for identifying potential functional impacts of cancer missense mutations and signs of positive selection across the patient cohort. 

## 2. Data Resources for Cancer Missense Mutations

The progress in this rapidly developing field has induced unprecedented growth in databases on genetic variants, such as cancer-oriented databases and databases storing different types of human genetic variations [18,19,20,21]. These databases provide important resources for detecting disease-causing or cancer-driving mutations and serve as the training templates or testing benchmarks for development of in silico prediction methods. Cancer genome sequencing projects have revealed vast numbers of somatic missense mutations in protein coding regions. The Cancer Genome Atlas (TCGA) was jointly supervised by the National Cancer Institute (NCI) and National Human Genome Research Institute (NHGRI) funded by National Institutes of Health (NIH), USA, in 2006 [1]. Since then, it has led to the characterization of key genomic changes in 33 cancer types that have improved evaluation of the biological relevance of genomic changes in cancer genomics discovery. As of 15 February 2018 (Data Release 10.1), TCGA contained 2,948,799 single base substitutions, 1,648,416 of which are missense variants. The International Cancer Genome Consortium (ICGC) was launched in 2008 by world-leading cancer and genomic researchers, aiming to describe systematically the genomic, transcriptomic and epigenomic abnormalities across 50 different cancer types or subtypes [2]. As of 7 December 2017 (Data Release 26), ICGC had collected 1,145,123 missense mutations out of 62,132,526 somatic substitutions from 20,383 donors, providing comprehensive insights into the landscape of somatic mutations and accelerating the discovery of cancer causes.

COSMIC is the world’s largest somatic cancer mutations repository database [18]. It includes not only mutations from patients whole-genome and -exome sequencing projects but also from cancer cell lines, which offers a most comprehensive resource for exploring the impact of somatic mutations in human cancer. However, not all cancer mutations provide a selective growth advantage to cancer cells. Large efforts dedicated to the detection of cancer driver mutations have yielded significant improvements in precision cancer medicine. In connection with this, several databases of cancer alterations were subsequently developed [22,23,24]. The Database of Curated Mutations (DoCM) is a public repository of disease-causing somatic cancer mutations comprehensively curated from literature with established relevance to cancer biology [25]. DoCM v3.2 includes 1364 variants and 1276 missense mutations from 122 cancer subtypes, enabling the cancer research community to aggregate, store, and track biologically important cancer variants that are essential for clinical annotation. Clinical Interpretations of Variants in Cancer (CIViC) is a community-edited web resource for discovering clinical interpretations of variants in cancer [26], which provides an educational forum for the dissemination of knowledge and active discussion of the clinical significance of cancer genome alterations. As of 22 February 2018, CIViC included 1767 variants, enabling precision medicine in cancer treatment. It should be mentioned that all these databases largely overlap in terms of their entries and may contain predictions as well as experimental validations mostly reporting potential driver mutations with a consistent lack of cancer somatic neutral variants.

In summary, the aforementioned data resources (Table 1) provide a variety of data for systematically exploring genomic, epigenomic and transcriptomic characteristics of tumor samples. These data not only allow for but also call for, the development of methods and tools that can efficiently detect cancer-related mutations and genes.

## 3. Computational Methods and Web Tools

### 3.1. 3D Spatial Distributions of Cancer Missense Mutations

Three-dimensional (3D) structures of proteins and their complexes could provide crucial information for identifying cancer-driving mutations. Thus, servers or databases for exploring and building the relationship between cancer-related missense mutations and structures will be useful for deciphering the biological consequences of these mutations (see Table 2). NCBI resources provide different platforms to map and analyze single nucleotide polymorphisms (SNPs) or cancer mutations with respect to protein structures (see the detailed description in [19]). In addition, the Cancer3D database helps users analyze the distribution of cancer somatic missense mutations from TCGA and CCLE (The Cancer Cell Line Encyclopedia project) in the context of 3D protein structures [29], allowing users to predict novel cancer drivers or drug biomarkers. dSysMap is a resource that maps disease/cancer-related mutations obtained from Uniprot in protein structure and interactions in the human interactome [30]. This program helps in rationalizing the mechanism of action for these mutations by putting them in a systemic context. The StructMAn server provides annotation of human and non-human non-synonymous single-nucleotide variants in a structural context [31]. It analyzes the spatial location of mutated sites in protein 3D structures relative to other binding partners of proteins, nucleic acids or low molecular-weight ligands. This tool provides structural context for up to 60% of nonsynonymous single-nucleotide variations (nsSNVs) in genes related to human diseases by searching for all structures of corresponding proteins and other homologs. 

Several methods have been recently developed for identifying cancer drivers using protein 3D structure information. Hotspot regions are demonstrated to have biological relevance in cancer. Kamburov et al. proposed a method to detect cancer genes using significant 3D clustering of mutations in the corresponding protein structure [57]. They applied this approach and analyzed pan-cancer somatic mutations from thousands of tumors falling within 18,356 proteins, among of them 5140 human proteins with known human protein 3D structures (51,980 3D structures). Eight well-established oncogenes (*PIK3CA*, *PTPN11, BRAF* and *HRAS*) and tumor suppressors (*PTEN*, *TP53, FBXW7 and CDKN2A*) with significant 3D clustering of missense mutations were detected. They concluded that systematic consideration of 3D structure can aid in identifying cancer genes with the understanding of the functional assignment of their mutations. For example, mutations that cluster at protein–protein interfaces may disturb key molecular interaction and function. Tokheim et al. also presented a novel and stringent algorithm using 3D protein structures to detect missense mutation hotspot regions in human cancer [58], enabling the discovery of hotspot regions in more genes. In addition to experimentally determined protein structures, they also considered high-quality structural models, so the genomic coverage increased from 5000 to more than 15,000 genes. This study can help cancer researchers investigate the biological functions of cancer somatic missense mutations by linking to the corresponding 3D protein structures. For example, the identified hotspot region in *RAC1* overlaps with the binding site. It contains a mutation in melanoma that has been identified as dysregulating *RAC1* by a fast cycling mechanism [59]. A computational tool, HotSpot3D, was developed by Niu et al. to identify protein 3D spatial hotspots (clusters) and to interpret the potential function of variants within them [60]. They applied HotSpot3D to more than 4000 TCGA tumors across 19 cancer types and discovered more than 6000 intra- and intermolecular clusters. In addition, they identified 369 rare mutations and 99 medium-recurrent mutations, all residing within clusters having potential functional implications. Furthermore, the predictions were validated in *EGFR* using high-throughput phosphorylation data and cell-line based experimental evaluation. Their mutation-drug cluster and network analysis predicted over 800 promising candidates for druggable mutations, providing new possibilities for designing personalized treatments.

### 3.2. Assessing Changes in Protein Conformation induced by Mutations 

The effects of mutations on macromolecular conformational dynamics are important [61]. Changes in macromolecular conformational dynamics, especially for proteins whose function is activated by conformational changes, can cause disease [62,63,64]. The effects of cancer mutations on oncogene conformations and functions have been studied extensively, both experimentally and computationally. L858R is an activating mutation in EGFR that is found in a large fraction of cancer patients. The mutant protein shows up to a 50-fold increase in activity compared to the wild type [65]. According to different proposed mechanisms, L858R mutation can either lock the kinase in the active state by preventing formation of the inactive state helical conformation [65] and/or it can reduce the intrinsic disorder content, favoring dimerization and stabilization of the active conformation [66]. Extensive molecular dynamics simulations with enhanced sampling demonstrated that L858R stabilizes the active conformation of EGFR more than the inactive conformation and rigidifies the αC-helix. Interestingly, the L858R and T790M double mutants exhibit significant positive epistasis [67,68]. 

Proteins may adopt different conformations during a biochemical reaction, and their intrinsic flexibility and ability to assume alternative conformations are crucial for protein function. Mutations might shift the equilibrium between different conformations and, as a result, the conformation of a mutated protein can differ in structure, stability and functional activity from the wild-type conformation. It is extremely difficult to model structural changes in a protein backbone produced by mutations. In fact, most algorithms discussed in the previous sections do not account for backbone flexibility. If several conformations are available in the structural databank for the same protein, ideally all of them should be used to provide a complete picture of dynamic and energetic mutational effects. 

All-atom molecular dynamics (MD) simulation is a commonly used approach to study bio-macromolecule conformational dynamics [69,70,71]. Using MD, one can simulate changes in conformations and hydrogen-bond networks [72,73,74,75,76,77]. Atomistic molecular dynamics simulation is based on Newton’s equations of motion, and the force is calculated by differentiating the potential energy with respect to the position of each atom in the system. The potential energy of the system is estimated based on a set of empirical parameters and equations, called a force field. The output of an MD simulation can be used to yield physical observations for a system, such as distances between atoms or residues, changes in hydrogen-bond networks, or secondary structures. The accuracy of MD simulation largely depends on the given 3D structures of the biomolecules. The current existing molecular dynamics packages and force fields have been rather successful in revealing these changes for mutations that do not induce dramatic structural alterations. The most widely used packages are NAMD [78], CHARMM [79] and Amber [80]. NAMD, for example, is fast and easy to use. It can be applied in conjunction with the CHARMM or Amber force fields. 

Mutations can either change the global conformation of an entire molecule or have a more localized effect. With respect to the effects of oncogenic mutations, for example, MD simulations and energy calculations were performed for the effects of several mutations from the same DNA-binding loop on the NFAT5 transcription factor [81]. Results illustrated that the effects of these mutations on protein conformations and binding with DNA were drastically different, although all mutations were located very close to each other in both sequence and structure. In particular, a phosphomimetic mutation, T222D, made the overall complex very rigid, whereas other mutations increased its flexibility. Demir et al. studied a variety of missense mutants by measuring their functional activity and thermodynamic stability [82]. In parallel, they performed molecular dynamics simulations for each mutant and calculated the number of distinct conformations in the dynamic landscape for measuring protein flexibility globally. They found that the number of individual protein conformations obtained from a simulation trajectory correlated well with thermodynamic stability and protein functional activity, indicating that mutants can lead to protein loss-of-function by increasing protein flexibility. 

### 3.3. Estimating the Effects of Mutations on Protein Stability

One can considerably decrease the number of potential cancer driver mutation candidates by determining the functional impact of each mutation on its corresponding protein. Protein stability may directly relate to functional activity, and changes in stability or incorrect folding could be major consequences of pathogenic missense mutations. It was previously shown that missense mutations destabilize tumor suppressors significantly more than SNPs, but this same effect was not observed for oncogenes [83]. In most cases, missense mutations are deleterious due to decreasing the stability of the corresponding protein [67,84]. For example, oncogenic mutations disrupt Casitas B-lineage lymphoma (CBL) function by decreasing the stability of CBL proteins [85]. Six mutations in the tumor suppressor gene phosphatase and tensin homolog (PTEN) in patients with PHTS-associated cancer show a global decrease in structural stability and increased dynamics across the domain interface [86]. In other cases, missense mutations may cause diseases by enhancing stability of the corresponding protein [87]. 

Computational methods that accurately predict the effects of variations on protein stability may help to identify functionally important mutations. Typically, the magnitude of mutational effects on stability can be quantified by unfolding free energy changes ∆∆*G_fold_*. The ProTherm database is a collection of thermodynamic parameters for wild-type and mutant proteins [27]. It includes unfolding Gibbs free energy, enthalpy and heat capacity changes, etc. that provide important clues for understanding the relationship among structure, stability and function of proteins and their mutants. This database also contains information on experimental conditions and methods used for measuring these data, which is frequently used as training templates for development of the following in silico prediction methods (Table 1).

Table 2 lists major computational approaches and tools for predicting quantitative changes in unfolding free energy in response to mutations. They are different in terms of algorithms used for training models, procedures used for optimization and sampling of protein conformations, and terms of energy functions. The terms of energy functions may vary from physics-based force fields to knowledge-based potentials by combining different structure-based or sequence-based physicochemical properties of amino acids. In addition, some methods take into account experimental conditions, such as salt concentration, pH values and temperature, which are important for assessing the free energy at near physiological conditions. For example, FoldX uses an empirical force field to evaluate the effects of mutations on stability, folding and dynamics in proteins and DNA [37]. One of the core functionalities of FoldX is the calculation of the unfolding free energy of a macromolecule based on its 3D structure. Its energy function is parametrized on experimental changes of unfolding free energy. FoldX is a software package, can be easily run on the Linux system, and allows users to deal with large datasets. FoldX has become a standard tool for predicting the effects of mutations including both single and multiple mutations on protein stability. SAAFEC is an approach that uses weighted MM-PBSA (Molecular Mechanics - Poisson-Boltzmann Surface Area) methods and various biophysical terms parametrized on thousands of experimental values [38]. Its energy terms are calculated using minimized wild-type and mutant structures. In particular, missing residues in the 3D structures can be added by SAAFEC. 

The majority of the above mentioned methods require coordinates of protein 3D structures as the inputs. Prediction accuracy can be influenced by different factors, including protein class and structural flexibility, type of substituted and wild type amino acid and structural environment of the substituted site. The performance of these predictors was assessed and compared in different studies using datasets of experimentally characterized mutants [88,89,90,91,92]. In the first study [92], the performance of six different methods were evaluated on a large set of 2156 single mutations, and the mutations used for training each model were excluded. The following performance ranking was reported: EGAD > CC/PBSA > I-Mutant2.0 > FoldX > Hunter > Rosetta with correlation coefficients between predicted and experimental ΔΔG values in the range of 0.59 and 0.26 and standard deviation in the range of 0.95 and 2.32 kcal mol^−1^. However, the servers, EGAD and CC/PBSA, with the top performances are no longer available. In the second study [91], 11 online stability predictors (CUPSAT, Dmutant, FoldX, I-Mutant2.0, two versions of I-Mutant3.0 (sequence and structure versions), MultiMutate, MUpro, SCide, Scpred, and SRide) were compared by performing a systematic analysis on 1784 single mutations excluding those used for training each program. I-Mutant3.0, Dmutant, and FoldX were found to be the most reliable predictors. Furthermore, Kepp evaluated the relative performance of these methods by calculating the stability changes of SOD1 and myoglobin variants [89,90]. Five methods, CUPSAT, I-Mutant2.0, I-Mutant3.0, PoPMuSiC and SDM, were tested on 54 SOD1 mutations. The results showed that PoPMuSiC was the most accurate approach with correlation coefficient R ~ 0.5 and MAE ~ 1.0 kcal mol^−1^ and followed by I-Mutant. Kumar et al. extended this study for SOD1 stability changes upon mutations using three different structures and four additional protein stability predictors (PoPMuSiC 3.1, FoldX, mCSM and ENCoM) [88]. Overall, PoPMuSiC and FoldX were shown as the best methods.

### 3.4. Estimating Quantitative Effects of Mutations on Protein–Protein or Protein–Nucleic Acid Interactions

A protein’s ability to establish highly selective interactions with macromolecular partners is a crucial prerequisite for proper biological function. A missense mutation affecting protein interactions [93,94,95] may cause significant perturbations or complete abolishment of protein function, potentially leading to disease. The binding free energy change ∆∆*G_bind_* is a way to quantify the magnitude mutational effects on protein-protein or protein-nucleic acid interactions. The SKEMPI database (Table 1) [28] includes experimentally measured values of change in thermodynamic parameters for binding affinity and kinetic rate constants upon single and multiple amino acid substitutions for protein-protein interactions with experimentally determined heterodimeric complex structures. It was derived from scientific literature and contains binding free energy, enthalpy and rate constant changes in response to mutations. The ProNIT database [27] is a collection of experimentally determined thermodynamic interaction parameters between proteins and nucleic acids, including binding constants, changes in free energy, enthalpy and heat capacity, with experimentally determined complex structures. These two databases were used as training benchmarks for development of the following prediction methods.

Table 2 lists several methods to estimate ∆∆*G_bind_* values. These methods require all-atom or at least protein backbone atom coordinates of a wild type. BeAtMuSiC, is a coarse-grained predictor of binding affinity changes in response to point mutations that uses different statistical potentials trained with known protein structures [49]. The BeAtMuSiC server provides an option for rapidly calculating the binding affinity changes for all possible mutations in a protein chain, while it does not make a model of the mutant structure. MutaBind is a web-based application method for evaluation of the effects of sequence variants and disease mutations on protein-protein interactions [48]. The MutaBind method relies on a combination of molecular mechanics force fields, statistical potentials and fast side-chain optimization algorithms. It can map mutations on a protein complex structure, calculate the associated changes in binding affinity, determine the deleterious effects of a mutation, estimate the confidence of this prediction and produce a mutant structure model for download. MutaBind was compared with BeAtMuSiC and FoldX by testing on two independent test sets and the results showed that MutaBind performs better than the other methods as evident from the values of correlation coefficients and root-mean-square errors. The MutaBind server was applied to estimate the putative changes in binding affinity of *Spalax* p53 interactions with other DDR proteins [96]. The calculated results supported the possibility that *Spalax’s* stress-related substitutions in TAD2 decrease the binding affinity of p53 to other DDR proteins as compared to humans. Another similar method, SAAMBE, is based on modified MM/PBSA-based components along with a set of statistical terms derived from physico-chemical properties of protein complexes [50,51]. 

Protein–protein interactions can be modulated by small-molecule drugs and biologics, such as peptides and antibodies. They are often considered druggable targets in anticancer therapy. As coverage of protein families with structural protein–protein interactions remains limited [97], integrative studies identifying key interactions in cancer pathways using protein structural similarity and homology to infer potential drug–protein interactions represent a promising data-driven strategy [98,99,100]. It is indeed instrumental and essential to have information about the locations of binding site residues on protein–protein interfaces, as well as binding specificity of interfaces with respect to interaction partners. 

There are very few methods available for predicting the effects of mutations on protein–nucleic acid interaction. mCSM-NA, for example, performs this task by relying on graph-based signatures that encode distance patterns between atoms [55]. mCSM-NA was trained on the entire ProNIT database and did not consider some special cases, such as the mismatch of nucleic acid sequences used in measuring binding affinity changes experimentally and in 3D protein–nucleic acid structures for developing the model. Another method, SAMPDI, uses a combination of modified MM/PBSA-based energy and knowledge-based terms to predict changes in binding affinity in response to mutations, in particular, for protein–DNA complexes [56]. SAMPDI was benchmarked against purged experimental data of protein–DNA interactions from the latest ProNIT database and data from the recent references. Compared with mCSM-NA, SAMPDI provides relative contribution of each energy term and additional structural information. For the majority of these methods, the rational choices of structure optimization protocols, energy terms or solvation models are determinants for achieving reasonable prediction accuracy. Moreover, prediction accuracy depends on the mutation type and its location in a protein–protein or protein–nucleic acid complex [101]. For example, interfacial mutations exhibit larger effects on protein–protein or protein–nucleic acid interactions compared to non-interfacial mutations [48,93,101,102]. Although available methods for structure modeling and analysis, energy calculations, assessment of conformational dynamics and functional annotations still need considerable improvement, they can provide meaningful results if they are applied correctly to the problems they aim to solve [62,67,84,103,104,105,106,107,108,109,110,111,112,113,114,115,116,117,118]. Herein, we present an example of the detailed analysis of cancer mutations molecular mechanisms for Casitas B-lineage lymphoma (CBL) protein activity [119]. The Cbl RING finger ubiquitin ligase (E3) plays both positive and negative regulatory roles in tyrosine kinase signaling and is aberrantly activated in many cancers. Oncogenic mutations in the *CBL* gene have been found in many tumors [85], but the mechanistic significance of these mutations and their impacts on CBL function were largely unknown [85,120]. Four CBL structures have been solved, representing snapshots of different stages of the CBL activation cycle. Computational modeling was applied to all four stages. First, cancer-related missense mutations for the *CBL* gene were extracted from the COSMIC database and mapped to all four CBL and CBL-E2 complex structures. All possible single-nucleotide substitutions resulting in amino acid changes in the *CBL* gene were produced as a reference set. Second, wild-type and mutant structures were optimized using a previously developed optimization protocol [101] that was performed with the NAMD program using the CHARMM27 force field [121]. Third, the unfolding free energy changes in response to mutations were calculated using the optimization procedure implemented in the FoldX program. Fourth, the binding free energy changes were calculated according to the previously introduced approach [101]. Finally, in vivo experiments of CBL-mediated EGFR ubiquitination for 15 mutations in three human cell lines were performed. The results indicated that computational approaches incorporating multiple protein conformations, stability, and binding affinity evaluations can successfully predict the magnitude of effects due to mutations and further help understand their mechanisms of action. 

### 3.5. Assessing Driver Status of Cancer Mutations

Many methods and tools have been developed over the past several years for predicting the functional impact of missense mutations [9,122], such as MutationAssessor [123] and PROVEAN [124]. These methods utilize a variety of features that describe the properties of a mutation from the aspects of evolutionary conservation, physicochemical attributes, or sequence context. Among them, several approaches are specifically designed for cancer missense mutations. The functional analysis through hidden Markov models (FATHMM)-cancer [125] is an algorithm that predicts the potential functional impact of cancer missense mutations. It uses cancer-associated mutations (germ line and somatic) from the CanProVar database [126] and putative neutral polymorphisms from the UniProt database as the training set and features of conservation and epigenomic signals. CHASM [127] is another approach for prioritizing cancer-driver mutations based on a random forest classifier [128,129] that was trained on 49 predictive features. The training set used for developing CHASM includes missense mutations from the COSMIC database and breast, colorectal, and pancreatic tumor resequencing studies [8,130,131,132]. Passenger mutations were synthetized by sampling from eight multinomial distributions that depend on dinucleotide context and tumor type. CHASM and other approaches focus on properties of individual mutations and does not explicitly rely on the frequency at which mutations appear in a gene, so it can potentially detect driver mutations occurring at low frequencies. In addition, CHASM is trained in a cancer-type-specific fashion and can be adapted to different cancer types. CanDrA [133] is a weighted supporting vector machine (SVM)-based tool for prioritizing somatic missense mutations by incorporating 95 structural and evolutionary features generated by over 10 functional prediction algorithms. Driver and passenger mutations selected based on the observed frequency for training of the model were taken from glioblastoma multiforme and ovarian carcinoma patients from COSMIC. They have precomputed CanDrA scores for almost all possible missense mutations across whole genome and allowed users to perform very efficient predictions. 

A recent systematic study was performed for comparing 15 such methods including FATHMM-cancer, CHASM and CanDrA that are introduced here on 849 non-neutral and 140 neutral mutations affecting 15 cancer genes. Cancer-specific mutation effect predictors display no-to-almost perfect agreement in their predictions of these SNVs and none of them were yet sufficiently reliable to guide high-cost experimental or clinical follow through [134]. ParsSNP [135] is an unsupervised functional impact predictor that uses an innovative, parsimony-based approach to prioritize cancer driver mutations. ParsSNP does not use predefined training labels that can introduce biases, but rather utilizes an expectation–maximization framework to find mutations that explain tumor incidence, so it can be applied to the problems that lack sufficient training samples for supervised methods. In particular, ParsSNP can identify truncation events in the tumor suppressor, while methods like CHARM and CanDrA are designed to work only with missense mutations. In their study, ParsSNP was reported to outperform the existing tools (CanDrA, CHASM and FATHMM Cancer) across five distinct benchmarks. In addition, the authors applied ParsSNP to an independent dataset of 30 patients with diffuse-type cancer, and ParsSNP identified many known and likely driver mutations that other methods did not detect. 

DNA context-dependent mutability is an important factor affecting frequencies at which cancer mutations reoccur in tumor samples [136]. Therefore, it is necessary to integrate context-dependent mutations into cancer-specific mutational models. To achieve this task, the MutaGene server (https://www.ncbi.nlm.nih.gov/research/mutagene/) provides tools for the analysis of expected mutability of mutations for cancer-specific and pan-cancer cases, ranking and predicting whether mutations are drivers or passengers [137,138]. 

This review attempts to outline the current development of computational approaches for prioritizing cancer driver missense mutations using various biophysical characteristics, including stability, binding affinity, and conformation dynamics. It was demonstrated that these biophysics-based approaches can identify functionally important missense mutations and facilitate understanding of the mechanisms of molecular effects in human cancer. In addition, we present a collection and introduction of the most comprehensive databases that store different types of sequencing data on cancer somatic missense mutations to the highly curated databases from the literature with established relevance to cancer biology and clinical annotation. It is important to emphasize that these approaches have limited capacity to identify driver mutations for tumor development directly. The reason for this is primarily that very few mutations have been validated as causative. Rather, they are able to prioritize candidates for follow-up experiments that may illustrate the actual physiological relevance of these mutations in cancer.

## Figures and Tables

**Table 1:** Summary of data resources for cancer somatic mutations and development of computational tools for predicting the effects of mutations on protein stability, protein–protein interaction and protein–nucleic acid interaction.

Name	Description	Web Site	Ref.
**Databases of cancer somatic mutations**			
COSMIC	Somatic mutations in cancer	http://cancer.sanger.ac.uk/cosmic	[18]
TCGA	Cancer Genome Atlas	http://cancergenome.nih.gov/	[1]
ICGC	International Cancer Genome Consortium	https://icgc.org	[2]
DOCM	A highly curated database of somatic mutations with characterized functional or clinical significance in cancer.	http://docm.genome.wustl.edu	[25]
CIViC	Provide supported clinical interpretations of cancer-related mutations	https://civic.genome.wustl.edu/home	[26]
**Databases of thermodynamic parameters**			
Protherm	Changes in thermodynamic parameters upon mutation for protein stability	http://gibk26.bse.kyutech.ac.jp/jouhou/Protherm/protherm.html	[27]
SKEMPI	Changes in thermodynamic parameters and kinetic rate constants upon mutation for protein-protein interactions	https://life.bsc.es/pid/mutation_database/	[28]
ProNIT	Changes in thermodynamic parameters upon mutation for protein-nucleic acid interactions	http://gibk26.bse.kyutech.ac.jp/jouhou/pronit/pronit.html	[27]

**Table 2:** A summary of online and free software resources for analyzing 3D spatial distribution of cancer missense mutations, predicting the effects of mutations on protein stability, protein-protein and protein-nucleic acid binding affinity. All resources need structure as an input except those with “*”.

Name	Description	Web Site	Ref.
**Analyzing 3D spatial distributions of cancer missense mutations**			
Cancer3D	Mapping somatic missense mutations from human proteins to protein structure from Protein Data Bank (PDB)	http://www.cancer3d.org	[29]
COSMIC-3D	Understanding cancer mutations in the context of 3D protein structure	https://cancer.sanger.ac.uk/cosmic3d/	[32]
cBioPortal	Visualization and analysis of large cancer studies. It is based on TCGA and incorporates the overlapping data from COSMIC	http://cbioportal.org/	[33,34]
dSysMap	The systematic mapping of disease-related missense mutations on the structurally annotated binary human interactome	https://dsysmap.irbbarcelona.org	[30]
MuPIT	Mapping the genomic coordinates of SNVs onto the 3D protein structures	http://mupit.icm.jhu.edu/MuPIT_Interactive/	[35]
StructMAn	Annotating nsSNVs in the context of the structural neighborhood of the resulting variations in the protein	http://structman.mpi-inf.mpg.de	[31]
SpacePAC	Identification of mutational clusters while considering protein tertiary structure	https://www.bioconductor.org/packages/release/bioc/html/SpacePAC.html	[36]
**Predicting protein stability changes upon mutations**			
FoldX	ΔΔG using empirical force fields	http://fold-x.embl-heidelberg.de	[37]
SAAFEC	ΔΔG using multiple linear regression	http://compbio.clemson.edu/SAAFEC/	[38]
mCSM	ΔΔG using graph-based signatures	http://biosig.unimelb.edu.au/mcsm/	[39]
CUPSAT	ΔΔG using mean force atom pair and torsion angle potentials	http://cupsat.tu-bs.de/	[40]
AUTO-MUTE	ΔΔG using knowledge-based potentials	http://proteins.gmu.edu/automute	[41]
NeEMO	ΔΔG using residue interaction networks	http://protein.bio.unipd.it/neemo/	[42]
MAESTRO	ΔΔG using multi agent stability prediction	http://biwww.che.sbg.ac.at/MAESTRO	[43]
ProMaya	ΔΔG using random forests regression	http://bental.tau.ac.il/ProMaya/	[44]
I-Mutant3.0 *	ΔΔG using SVMs	http://gpcr2.biocomp.unibo.it/cgi/predictors/I-Mutant3.0/I-Mutant3.0.cgi	[45]
MUPro *	Predicts qualitative decrease/increase of stability using SVM	http://mupro.proteomics.ics.uci.edu/	[46]
iStable *	ΔΔG using SVM	http://predictor.nchu.edu.tw/iStable	[47]
**Predicting protein-protein binding affinity changes upon mutations**			
MutaBind	ΔΔG using molecular mechanics force fields, statistical potentials and fast side-chain optimization algorithms built via multiple linear regression and random forest	https://www.ncbi.nlm.nih.gov/research/mutabind/	[48]
BeAtMuSiC	ΔΔG using a set of statistical potentials	http://babylone.ulb.ac.be/beatmusic	[49]
SAAMBE	ΔΔG using modified MM-PBSA based energy terms and a set of statistical terms built via multiple linear regression	http://compbio.clemson.edu/saambe_webserver/	[50,51]
BindProf	ΔΔG using structure-based interface profiles	https://zhanglab.ccmb.med.umich.edu/BindProf/	[52]
DrugScore^PPI^	ΔΔG for alanine-scanning mutations located on interface using knowledge-based scoring functions	http://cpclab.uni-duesseldorf.de/dsppi/	[53]
SNP-IN	A classifier of effects on protein-protein interactions using supervised and semi-supervised learning	http://korkinlab.org/snpintool/	[54]
**Predicting protein-nucleic acid binding affinity changes upon mutations**			
mCSM-NA	ΔΔG relying on graph-based signatures and can predict the effects of single mutations on protein-nucleic acid binding	http://biosig.unimelb.edu.au/mcsm_na/	[55]
SAMPDI	ΔΔG combining modified MM-PBSA based energy terms with knowledge based terms for predicting the protein-DNA binding affinity changes upon single mutations	http://compbio.clemson.edu/SAMPDI/	[56]

## References

[1] Weinstein J.N., Collisson E.A., Mills G.B., Shaw K.M., Ozenberger B.A., Ellrott K., Shmulevich I., Sander C., Stuart J.M., Cancer Genome Atlas Research Network (2013). The Cancer Genome Atlas Pan-Cancer analysis project. Nat. Genet..

[2] Hudson T.J., Anderson W., Artez A., Barker A.D., Bell C., Bernabé R.R., Bhan M.K., Calvo F., Eerola I., Gerhard D.S. (2010). International network of cancer genome projects. Nature.

[3] Greenman C., Stephens P., Smith R., Dalgliesh G.L., Hunter C., Bignell G., Davies H., Teague J., Butler A., Stevens C. (2007). Patterns of somatic mutation in human cancer genomes. Nature.

[4] Fearon E.R., Vogelstein B. (1990). A Genetic Model for Colorectal Tumorigenesis. Cell.

[5] Tabin C.J., Bradley S.M., Bargmann C.I., Weinberg R.A., Papageorge A.G., Scolnick E.M., Dhar R., Lowy D.R., Chang E.H. (1982). Mechanism of Activation of a Human Oncogene. Nature.

[6] Chapman P.B., Hauschild A., Robert C., Haanen J.B., Ascierto P., Larkin J., Dummer R., Garbe C., Testori A., Maio M. (2011). Improved Survival with Vemurafenib in Melanoma with BRAF V600E Mutation. N. Engl. J. Med..

[7] Karapetis C.S., Khambata-Ford S., Jonker D.J., O'Callaghan C.J., Tu D., Tebbutt N.C., Simes R.J., Chalchal H., Shapiro J.D., Robitaille S. (2008). K-ras mutations and benefit from cetuximab in advanced colorectal cancer. N. Engl. J. Med..

[8] Wood L.D., Parsons D.W., Jones S., Lin J., Sjoblom T., Leary R.J., Shen D., Boca S.M., Barber T., Ptak J. (2007). The genomic landscapes of human breast and colorectal cancers. Science.

[9] Cheng F.X., Zhao J.F., Zhao Z.M. (2016). Advances in computational approaches for prioritizing driver mutations and significantly mutated genes in cancer genomes. Brief. Bioinform..

[10] Porta-Pardo E., Kamburov A., Tamborero D., Pons T., Grases D., Valencia A., Lopez-Bigas N., Getz G., Godzik A. (2017). Comparison of algorithms for the detection of cancer drivers at subgene resolution. Nat. Methods.

[11] Nussinov R., Tsai C.J. (2015). ‘Latent drivers’ expand the cancer mutational landscape. Curr. Opin. Struct. Biol..

[12] Leedham S., Tomlinson I. (2012). The Continuum Model of Selection in Human Tumors: General Paradigm or Niche Product?. Cancer Res..

[13] Krutovskikh V., Mazzoleni G., Mironov N., Omori Y., Aguelon A.M., Mesnil M., Berger F., Partensky C., Yamasaki H. (1994). Altered homologous and heterologous gap-junctional intercellular communication in primary human liver tumors associated with aberrant protein localization but not gene mutation of connexin 32. Int. J. Cancer.

[14] Chen Y., Chen C.F., Riley D.J., Allred D.C., Chen P.L., Von Hoff D., Osborne C.K., Lee W.H. (1995). Aberrant subcellular localization of BRCA1 in breast cancer. Science.

[15] Hung M.C., Link W. (2011). Protein localization in disease and therapy. J. Cell Sci..

[16] Wan S., Mak M.W., Kung S.Y. (2014). R3P-Loc: A compact multi-label predictor using ridge regression and random projection for protein subcellular localization. J. Theor. Biol..

[17] Wan S., Mak M.W., Kung S.Y. (2014). HybridGO-Loc: Mining hybrid features on gene ontology for predicting subcellular localization of multi-location proteins. PLoS ONE.

[18] Forbes S.A., Bindal N., Bamford S., Cole C., Kok C.Y., Beare D., Jia M., Shepherd R., Leung K., Menzies A. (2011). COSMIC: Mining complete cancer genomes in the Catalogue of Somatic Mutations in Cancer. Nucleic Acids Res..

[19] Li M., Goncearenco A., Panchenko A.R. (2017). Annotating Mutational Effects on Proteins and Protein Interactions: Designing Novel and Revisiting Existing Protocols. Methods Mol. Biol..

[20] Landrum M.J., Lee J.M., Riley G.R., Jang W., Rubinstein W.S., Church D.M., Maglott D.R. (2014). ClinVar: Public archive of relationships among sequence variation and human phenotype. Nucleic Acids Res..

[21] Stenson P.D., Mort M., Ball E.V., Shaw K., Phillips A., Cooper D.N. (2014). The Human Gene Mutation Database: Building a comprehensive mutation repository for clinical and molecular genetics, diagnostic testing and personalized genomic medicine. Hum. Genet..

[22] Tamborero D., Rubio-Perez C., Deu-Pons J., Schroeder M.P., Vivancos A., Rovira A., Tusquets I., Albanell J., Rodon J., Tabernero J. (2018). Cancer Genome Interpreter annotates the biological and clinical relevance of tumor alterations. Genome Med..

[23] Simonetti F.L., Tornador C., Nabau-Moreto N., Molina-Vila M.A., Marino-Buslje C. (2014). Kin-Driver: A database of driver mutations in protein kinases. Database.

[24] MacConaill L.E., Garcia E., Shivdasani P., Ducar M., Adusumilli R., Breneiser M., Byrne M., Chung L., Conneely J., Crosby L. (2014). Prospective Enterprise-Level Molecular Genotyping of a Cohort of Cancer Patients. J. Mol. Diagn..

[25] Ainscough B.J., Griffith M., Coffman A.C., Wagner A.H., Kunisaki J., Choudhary M.N., McMichael J.F., Fulton R.S., Wilson R.K., Griffith O.L. (2016). DoCM: A database of curated mutations in cancer. Nat. Methods.

[26] Griffith M., Spies N.C., Krysiak K., McMichael J.F., Coffman A.C., Danos A.M., Ainscough B.J., Ramirez C.A., Rieke D.T., Kujan L. (2017). CIViC is a community knowledgebase for expert crowdsourcing the clinical interpretation of variants in cancer. Nat. Genet..

[27] Kumar M.D.S., Bava K.A., Gromiha M.M., Prabakaran P., Kitajima K., Uedaira H., Sarai A. (2006). ProTherm and ProNIT: Thermodynamic databases for proteins and protein-nucleic acid interactions. Nucleic Acids Res..

[28] Moal I.H., Fernandez-Recio J. (2012). SKEMPI: A Structural Kinetic and Energetic database of Mutant Protein Interactions and its use in empirical models. Bioinformatics.

[29] Porta-Pardo E., Hrabe T., Godzik A. (2015). Cancer3D: Understanding cancer mutations through protein structures. Nucleic Acids Res..

[30] Mosca R., Tenorio-Laranga J., Olivella R., Alcalde V., Ceol A., Soler-Lopez M., Aloy P. (2015). dSysMap: Exploring the edgetic role of disease mutations. Nat. Methods.

[31] Gress A., Ramensky V., Buch J., Keller A., Kalinina O.V. (2016). StructMAn: Annotation of single-nucleotide polymorphisms in the structural context. Nucleic Acids Res..

[32] Harper K. (2017). Modeling Cancer Mutations in 3-D. Cancer Discov..

[33] Cerami E., Gao J., Dogrusoz U., Gross B.E., Sumer S.O., Aksoy B.A., Jacobsen A., Byrne C.J., Heuer M.L., Larsson E. (2012). The cBio Cancer Genomics Portal: An Open Platform for Exploring Multidimensional Cancer Genomics Data. Cancer Discov..

[34] Gao J.J., Aksoy B.A., Dogrusoz U., Dresdner G., Gross B., Sumer S.O., Sun Y.C., Jacobsen A., Sinha R., Larsson E. (2013). Integrative Analysis of Complex Cancer Genomics and Clinical Profiles Using the cBioPortal. Sci. Signal..

[35] Niknafs N., Kim D., Kim R., Diekhans M., Ryan M., Stenson P.D., Cooper D.N., Karchin R. (2013). MuPIT interactive: Webserver for mapping variant positions to annotated, interactive 3D structures. Hum. Genet..

[36] Ryslik G.A., Cheng Y.W., Cheung K.H., Bjornson R.D., Zelterman D., Modis Y., Zhao H.Y. (2014). A spatial simulation approach to account for protein structure when identifying non-random somatic mutations. BMC Bioinform..

[37] Guerois R., Nielsen J.E., Serrano L. (2002). Predicting changes in the stability of proteins and protein complexes: A study of more than 1000 mutations. J. Mol. Biol..

[38] Getov I., Petukh M., Alexov E. (2016). SAAFEC: Predicting the Effect of Single Point Mutations on Protein Folding Free Energy Using a Knowledge-Modified MM/PBSA Approach. Int. J. Mol. Sci..

[39] Pires D.E.V., Ascher D.B. (2014). Blundell, T.L. mCSM: Predicting the effects of mutations in proteins using graph-based signatures. Bioinformatics.

[40] Parthiban V., Gromiha M.M., Schomburg D. (2006). CUPSAT: Prediction of protein stability upon point mutations. Nucleic Acids Res..

[41] Masso M., Vaisman I.I. (2010). AUTO-MUTE: Web-based tools for predicting stability changes in proteins due to single amino acid replacements. Protein Eng. Des. Sel..

[42] Giollo M., Martin A.J.M., Walsh I., Ferrari C., Tosatto S.C.E. (2014). NeEMO: A method using residue interaction networks to improve prediction of protein stability upon mutation. BMC Genom..

[43] Laimer J., Hofer H., Fritz M., Wegenkittl S., Lackner P. (2015). MAESTRO—Multi agent stability prediction upon point mutations. BMC Bioinform..

[44] Wainreb G., Wolf L., Ashkenazy H., Dehouck Y., Ben-Tal N. (2011). Protein stability: A single recorded mutation aids in predicting the effects of other mutations in the same amino acid site. Bioinformatics.

[45] Capriotti E., Fariselli P., Rossi I., Casadio R. (2008). A three-state prediction of single point mutations on protein stability changes. Bmc Bioinformatics.

[46] Cheng J.L., Randall A., Baldi P. (2006). Prediction of protein stability changes for single-site mutations using support vector machines. Proteins Struct. Funct. Bioinform..

[47] Chen C.W., Lin J., Chu Y.W. (2013). iStable: Off-the-shelf predictor integration for predicting protein stability changes. BMC Bioinform..

[48] Li M., Simonetti F.L., Goncearenco A., Panchenko A.R. (2016). MutaBind estimates and interprets the effects of sequence variants on protein-protein interactions. Nucleic Acids Res..

[49] Dehouck Y., Kwasigroch J.M., Rooman M., Gilis M. (2013). BeAtMuSiC: Prediction of changes in protein-protein binding affinity on mutations. Nucleic Acids Res..

[50] Petukh M., Li M., Alexov E. (2015). Predicting Binding Free Energy Change Caused by Point Mutations with Knowledge-Modified MM/PBSA Method. PLoS Comput. Biol..

[51] Petukh M., Dai L., Alexov E. (2016). SAAMBE: Webserver to Predict the Charge of Binding Free Energy Caused by Amino Acids Mutations. Int. J. Mol. Sci..

[52] Brender J.R., Zhang Y. (2015). Predicting the Effect of Mutations on Protein-Protein Binding Interactions through Structure-Based Interface Profiles. PLoS Comput. Biol..

[53] Kruger D.M., Gohlke H. (2010). DrugScorePPI webserver: Fast and accurate in silico alanine scanning for scoring protein-protein interactions. Nucleic Acids Res..

[54] Zhao N., Han J.G., Shyu C.R., Korkin D. (2014). Determining Effects of Non-synonymous SNPs on Protein-Protein Interactions using Supervised and Semi-supervised Learning. PLoS Comput. Biol..

[55] Pires D.E.V., Ascher D.B. (2017). mCSM-NA: Predicting the effects of mutations on protein-nucleic acids interactions. Nucleic Acids Res..

[56] Peng Y.H., Sun L.X., Jia Z., Li L., Alexov E. (2018). Predicting protein-DNA binding free energy change upon missense mutations using modified MM/PBSA approach: SAMPDI webserver. Bioinformatics.

[57] Kamburov A., Lawrence M.S., Polak P., Leshchiner I., Lage K., Golub T.R., Lander E.S., Getz G. (2015). Comprehensive assessment of cancer missense mutation clustering in protein structures. Proc. Natl. Acad. Sci. USA.

[58] Tokheim C., Bhattacharya R., Niknafs N., Gygax D.M., Kim R., Ryan M., Masica D.L., Karchin R. (2016). Exome-Scale Discovery of Hotspot Mutation Regions in Human Cancer Using 3D. Protein Struct. Cancer Res..

[59] Davis M.J., Ha B.H., Holman E.C., Halaban R., Schlessinger J., Boggon T.J. (2013). RAC1P29S is a spontaneously activating cancer-associated GTPase. Proc. Natl. Acad. Sci. USA.

[60] Niu B., Scott A.D., Sengupta S., Bailey M.H., Batra P., Ning J., Wyczalkowski M.A., Liang W.W., Zhang Q., McLellan M.D. (2016). Protein-structure-guided discovery of functional mutations across 19 cancer types. Nat. Genet..

[61] Friedman R., Boye K., Flatmark K. (2013). Molecular modelling and simulations in cancer research. Biochim. Biophys. Acta Rev. Cancer.

[62] Takano K., Liu D., Tarpey P., Gallant E., Lam A., Witham S., Alexov E., Chaubey A., Stevenson R.E., Schwartz C.E. (2012). An X-linked channelopathy with cardiomegaly due to a CLIC2 mutation enhancing ryanodine receptor channel activity. Hum. Mol. Genet..

[63] Witham S., Takano K., Schwartz C., Alexov E. (2011). A missense mutation in CLIC2 associated with intellectual disability is predicted by in silico modeling to affect protein stability and dynamics. Proteins Struct. Funct. Bioinform..

[64] Tsukamoto H., Farrens D.L. (2013). A Constitutively Activating Mutation Alters the Dynamics and Energetics of a Key Conformational Change in a Ligand-free G Protein-coupled Receptor. J. Biol. Chem..

[65] Zhang X.W., Gureasko J., Shen K., Cole P.A., Kuriyan J. (2006). An allosteric mechanism for activation of the kinase domain of epidermal growth factor receptor. Cell.

[66] Shan Y.B., Eastwood M.P., Zhang X.W., Kim E.T., Arkhipov A., Dror R.O., Jumper J., Kuriyan J., Shaw D.E. (2012). Oncogenic Mutations Counteract Intrinsic Disorder in the EGFR Kinase and Promote Receptor Dimerization. Cell.

[67] Hashimoto K., Rogozin I.B., Panchenko A.R. (2012). Oncogenic potential is related to activating effect of cancer single and double somatic mutations in receptor tyrosine kinases. Hum. Mutat..

[68] Sutto L., Gervasio F.L. (2013). Effects of oncogenic mutations on the conformational free-energy landscape of EGFR kinase. Proc. Natl. Acad. Sci. USA.

[69] Salsbury F.R. (2010). Molecular dynamics simulations of protein dynamics and their relevance to drug discovery. Curr. Opin. Pharmacol..

[70] Zwier M.C., Chong L.T. (2010). Reaching biological timescales with all-atom molecular dynamics simulations. Curr. Opin. Pharmacol..

[71] Scheraga H.A., Khalili M., Liwo A. (2007). Protein-folding dynamics: Overview of molecular simulation techniques. Annu. Rev. Phys. Chem..

[72] Li M.H., Zheng W.J. (2013). All-Atom Molecular Dynamics Simulations of Actin-Myosin Interactions: A Comparative Study of Cardiac alpha Myosin, beta Myosin, and Fast Skeletal Muscle Myosin. Biochemistry.

[73] Li M.H., Zheng W.J. (2012). All-Atom Structural Investigation of Kinesin-Microtubule Complex Constrained by High-Quality Cryo-Electron-Microscopy Maps. Biochemistry.

[74] Li M.H., Zheng W.J. (2011). Probing the Structural and Energetic Basis of Kinesin-Microtubule Binding Using Computational Alanine-Scanning Mutagenesis. Biochemistry.

[75] Li M.H., Luo Q.A., Xue X.G., Li Z.S. (2011). Molecular dynamics studies of the 3D structure and planar ligand binding of a quadruplex dimer. J. Mol. Model..

[76] Li M.H., Luo Q., Li Z.S. (2010). Molecular Dynamics Study on the Interactions of Porphyrin with Two Antiparallel Human Telomeric Quadruplexes. J. Phys. Chem. B.

[77] Li M.H., Zhou Y.H., Luo Q., Li Z.S. (2010). The 3D structures of G-Quadruplexes of HIV-1 integrase inhibitors: Molecular dynamics simulations in aqueous solution and in the gas phase. J. Mol. Model..

[78] Phillips J.C., Braun R., Wang W., Gumbart J., Tajkhorshid E., Villa E., Chipot C., Skeel R.D., Kale L., Schulten K. (2005). Scalable molecular dynamics with NAMD. J. Comput. Chem..

[79] Brooks B.R., Bruccoleri R.E., Olafson B.D., States D.J., Swaminathan S., Karplus M. (1983). Charmm—A Program for Macromolecular Energy, Minimization, and Dynamics Calculations. J. Comput. Chem..

[80] Case D.A., Cheatham T.E., Darden T., Gohlke H., Luo R., Merz K.M., Onufriev A., Simmerling C., Wang B., Woods R.J. (2005). The Amber biomolecular simulation programs. J. Comput. Chem..

[81] Li M.H., Shoemaker B.A., Thangudu R.R., Ferraris J.D., Burg M.B., Panchenko A.R. (2013). Mutations in DNA-Binding Loop of NFAT5 Transcription Factor Produce Unique Outcomes on Protein-DNA Binding and Dynamics. J. Phys. Chem. B.

[82] Demir O., Baronio R., Salehi F., Wassman C.D., Hall L., Hatfield G.W., Chamberlin R., Kaiser P., Lathrop R.H., Amaro R.E. (2011). Ensemble-Based Computational Approach Discriminates Functional Activity of p53 Cancer and Rescue Mutants. PLoS Comput. Biol..

[83] Stehr H., Jang S.H.J., Duarte J.M., Wierling C., Lehrach H., Lappe M., Lange B.M.H. (2011). The structural impact of cancer-associated missense mutations in oncogenes and tumor suppressors. Mol. Cancer.

[84] Peng Y.H., Norris J., Schwartz C., Alexov E. (2016). Revealing the Effects of Missense Mutations Causing Snyder-Robinson Syndrome on the Stability and Dimerization of Spermine Synthase. Int. J. Mol. Sci..

[85] Kales S.C., Ryan P.E., Nau M.M., Lipkowitz S. (2010). Cbl and Human Myeloid Neoplasms: The Cbl Oncogene Comes of Age. Cancer Res..

[86] Smith I.N., Thacker S., Jaini R., Eng C. (2018). Dynamics and structural stability effects of germline PTEN mutations associated with cancer versus autism phenotypes. J. Biomol. Struct. Dyn..

[87] Chiang C.H., Grauffel C., Wu L.S., Kuo P.H., Doudeva L.G., Lim C., Shen C.K., Yuan H.S. (2016). Structural analysis of disease-related TDP-43 D169G mutation: Linking enhanced stability and caspase cleavage efficiency to protein accumulation. Sci. Rep..

[88] Kumar V., Rahman S., Choudhry H., Zamzami M.A., Sarwar Jamal M., Islam A., Ahmad F., Hassan M.I. (2017). Computing disease-linked SOD1 mutations: Deciphering protein stability and patient-phenotype relations. Sci. Rep..

[89] Kepp K.P. (2015). Towards a “Golden Standard” for computing globin stability: Stability and structure sensitivity of myoglobin mutants. Biochim. Biophys. Acta.

[90] Kepp K.P. (2014). Computing stability effects of mutations in human superoxide dismutase 1. J. Phys. Chem. B.

[91] Khan S., Vihinen M. (2010). Performance of protein stability predictors. Hum. Mutat..

[92] Potapov V., Cohen M., Schreiber G. (2009). Assessing computational methods for predicting protein stability upon mutation: Good on average but not in the details. Protein Eng. Des. Sel..

[93] Nishi H., Tyagi M., Teng S.L., Shoemaker B.A., Hashimoto K., Alexov E., Wuchty S., Panchenko A.R. (2013). Cancer Missense Mutations Alter Binding Properties of Proteins and Their Interaction Networks. PLoS ONE.

[94] Teng S.L., Madej T., Panchenko A., Alexov E. (2009). Modeling effects of human single nucleotide polymorphisms on protein-protein interactions. Biophys. J..

[95] Ghersi D., Singh M. (2014). Interaction-based discovery of functionally important genes in cancers. Nucleic Acids Res..

[96] Domankevich V., Opatowsky Y., Malik A., Korol A.B., Frenkel Z., Manov I., Avivi A., Shams I. (2016). Adaptive patterns in the p53 protein sequence of the hypoxia- and cancer-tolerant blind mole rat Spalax. BMC Evol. Biol..

[97] Goncearenco A., Shoemaker B.A., Zhang D.C., Sarychey A., Panchenko A.R. (2014). Coverage of protein domain families with structural protein-protein interactions: Current progress and future trends. Prog. Biophys. Mol. Biol..

[98] Shoemaker B.A., Zhang D.C., Tyagi M., Thangudu R.R., Fong J.H., Marchler-Bauer A., Bryant S.H., Madej T., Panchenko A.R. (2012). IBIS (Inferred Biomolecular Interaction Server) reports, predicts and integrates multiple types of conserved interactions for proteins. Nucleic Acids Res..

[99] Goncearenco A., Li M., Simonetti F.L., Shoemaker B.A., Panchenko A.R. (2017). Exploring Protein-Protein Interactions as Drug Targets for Anti-cancer Therapy with In Silico Workflows. Methods Mol. Biol..

[100] Acuner-Ozbabacan E.S., Engin B.H., Guven-Maiorov E., Kuzu G., Muratcioglu S., Baspinar A., Chen Z., Van Waes C., Gursoy A., Keskin O. (2014). The structural network of Interleukin-10 and its implications in inflammation and cancer. BMC Genom..

[101] Li M., Petukh M., Alexov E., Panchenko A.R. (2014). Predicting the Impact of Missense Mutations on Protein-Protein Binding Affinity. J. Chem. Theory Comput..

[102] David A., Sternberg M.J.E. (2015). The Contribution of Missense Mutations in Core and Rim Residues of Protein-Protein Interfaces to Human Disease. J. Mol. Biol..

[103] Zhang Z., Norris J., Kalscheuer V., Wood T., Wang L., Schwartz C., Alexov E., Van Esch H. (2013). A Y328C missense mutation in spermine synthase causes a mild form of Snyder-Robinson syndrome. Hum. Mol. Genet..

[104] Kucukkal T.G., Alexov E. (2015). Structural, Dynamical, and Energetical Consequences of Rett Syndrome Mutation R133C in MeCP2. Comput. Math. Methods Med..

[105] Zhang Z., Witham S., Petukh M., Moroy G., Miteva M., Ikeguchi Y., Alexov E. (2013). A rational free energy-based approach to understanding and targeting disease-causing missense mutations. J. Am. Med. Inform. Assoc..

[106] Zhang Z., Wang L., Gao Y., Zhang J., Zhenirovskyy M., Alexov E. (2012). Predicting folding free energy changes upon single point mutations. Bioinformatics.

[107] Petukh M., Kucukkal T.G., Alexov E. (2015). On Human Disease-Causing Amino Acid Variants: Statistical Study of Sequence and Structural Patterns. Hum. Mutat..

[108] Dolzhanskaya N., Gonzalez M.A., Sperziani F., Stefl S., Messing J., Wen G.Y., Alexov E., Zuchner S., Velinov M. (2014). A Novel p.Leu(381)Phe Mutation in Presenilin 1 is Associated with Very Early Onset and Unusually Fast Progressing Dementia as well as Lysosomal Inclusions Typically Seen in Kufs Disease. J. Alzheimers Dis..

[109] Boccuto L., Aoki K., Flanagan-Steet H., Chen C.F., Fan X., Bartel F., Petukh M., Pittman A., Saul R., Chaubey A. (2014). A mutation in a ganglioside biosynthetic enzyme, ST3GAL5, results in salt & pepper syndrome, a neurocutaneous disorder with altered glycolipid and glycoprotein glycosylation. Hum. Mol. Genet..

[110] Peng Y.H., Alexov E. (2016). Investigating the linkage between disease-causing amino acid variants and their effect on protein stability and binding. Proteins Struct. Funct. Bioinform..

[111] Gilis D., McLennan H.R., Dehouck Y., Cabrita L.D., Rooman M., Bottomley S.P. (2003). In vitro and in silico design of alpha1-antitrypsin mutants with different conformational stabilities. J. Mol. Biol..

[112] Zhang Z., Norris J., Schwartz C., Alexov E. (2011). In Silico and In Vitro Investigations of the Mutability of Disease-Causing Missense Mutation Sites in Spermine Synthase. PLoS ONE.

[113] Zhang S.B., Wu Z.L. (2011). Identification of amino acid residues responsible for increased thermostability of feruloyl esterase A from Aspergillus niger using the PoPMuSiC algorithm. Bioresour. Technol..

[114] Li L., Jia Z., Peng Y.H., Godar S., Getov I., Teng S.L., Alper J., Alexov E. (2017). Forces and Disease: Electrostatic force differences caused by mutations in kinesin motor domains can distinguish between disease-causing and non-disease-causing mutations. Sci. Rep..

[115] Zhang Z., Zheng Y.L., Petukh M., Pegg A., Ikeguchi Y., Alexov E. (2013). Enhancing Human Spermine Synthase Activity by Engineered Mutations. PLoS Comput. Biol..

[116] Li L., Chakravorty A., Alexov E. (2017). DelPhiForce, a Tool for Electrostatic Force Calculations: Applications to Macromolecular Binding. J. Comput. Chem..

[117] Peng Y.H., Alexov E. (2017). Computational investigation of proton transfer, pKa shifts and pH-optimum of protein-DNA and protein-RNA complexes. Proteins Struct. Funct. Bioinform..

[118] Zhang Z., Teng S.L. (2010). Wang, L.J.; Schwartz, C.E.; Alexov, E.; Computational Analysis of Missense Mutations Causing Snyder-Robinson Syndrome. Hum. Mutat..

[119] Li M.H., Kales S.C., Ma K., Shoemaker B.A., Crespo-Barreto J., Cangelosi A.L., Lipkowitz S., Panchenko A.R. (2016). Balancing Protein Stability and Activity in Cancer: A New Approach for Identifying Driver Mutations Affecting CBL Ubiquitin Ligase Activation. Cancer Res..

[120] Naramura M., Nadeau S., Mohapatra B., Ahmad G., Mukhopadhyay C., Sattler M., Raja S.M., Natarajan A., Band V., Band H. (2011). Mutant Cbl proteins as oncogenic drivers in myeloproliferative disorders. Oncotarget.

[121] MacKerell A.D., Bashford D., Bellott M., Dunbrack R.L., Evanseck J.D., Field M.J., Fischer S., Gao J., Guo H., Ha S. (1998). All-atom empirical potential for molecular modeling and dynamics studies of proteins. J. Phys. Chem. B.

[122] Gonzalez-Perez A., Mustonen V., Reva B., Ritchie G.R.S., Creixell P., Karchin R., Vazquez M., Fink J.L., Kassahn K.S., Pearson J.V. (2013). Computational approaches to identify functional genetic variants in cancer genomes. Nat. Methods.

[123] Reva B., Antipin Y., Sander C. (2011). Predicting the functional impact of protein mutations: Application to cancer genomics. Nucleic Acids Res..

[124] Choi Y., Sims G.E., Murphy S., Miller J.R., Chan A.P. (2012). Predicting the functional effect of amino acid substitutions and indels. PLoS ONE.

[125] Shihab H.A., Gough J., Cooper D.N., Day I.N.M., Gaunt T.R. (2013). Predicting the functional consequences of cancer-associated amino acid substitutions. Bioinformatics.

[126] Li J., Duncan D.T., Zhang B. (2010). CanProVar: A human cancer proteome variation database. Hum. Mutat..

[127] Carter H., Chen S.N., Isik L., Tyekucheva S., Velculescu V.E., Kinzler K.W., Vogelstein B., Karchin R. (2009). Cancer-specific high-throughput annotation of somatic mutations: Computational prediction of driver missense mutations. Cancer Res..

[128] Breiman L. (2001). Random forests. Mach. Learn..

[129] Amit Y., Geman D. (1997). Shape quantization and recognition with randomized trees. Neural Comput..

[130] Jones S., Zhang X.S., Parsons D.W., Lin J.C.H., Leary R.J., Angenendt P., Mankoo P., Carter H., Kamiyama H., Jimeno A. (2008). Core signaling pathways in human pancreatic cancers revealed by global genomic analyses. Science.

[131] Parsons D.W., Jones S., Zhang X.S., Lin J.C.H., Leary R.J., Angenendt P., Mankoo P., Carter H., Siu I.M., Gallia G.L. (2008). An integrated genomic analysis of human glioblastoma multiforme. Science.

[132] Sjoblom T., Jones S., Wood L.D., Parsons D.W., Lin J., Barber T.D., Mandelker D., Leary R.J., Ptak J., Silliman N. (2006). The consensus coding sequences of human breast and colorectal cancers. Science.

[133] Mao Y., Chen H., Liang H., Meric-Bernstam F., Mills G.B., Chen K. (2013). CanDrA: Cancer-specific driver missense mutation annotation with optimized features. PLoS ONE.

[134] Martelotto L.G., Ng C.K.Y., De Filippo M.R., Zhang Y., Piscuoglio S., Lim R.S., Shen R.L., Norton L., Reis-Filho J.S., Weigelt B. (2014). Benchmarking mutation effect prediction algorithms using functionally validated cancer-related missense mutations. Genome Biol..

[135] Kumar R.D., Swamidass S.J., Bose R. (2016). Unsupervised detection of cancer driver mutations with parsimony-guided learning. Nat. Genet..

[136] Nik-Zainal S., Alexandrov L.B., Wedge D.C., Van Loo P., Greenman C.D., Raine K., Jones D., Hinton J., Marshall J., Stebbings L.A. (2012). Mutational processes molding the genomes of 21 breast cancers. Cell.

[137] Goncearenco A., Rager S.L., Li M.H., Sang Q.X., Rogozin I.B., Panchenko A.R. (2017). Exploring background mutational processes to decipher cancer genetic heterogeneity. Nucleic Acids Res..

[138] Li M., Brown A.-L., Goncearenco A., Panchenko A.R. (2018). Nucleotide and codon background mutability shape cancer mutational spectrum and advance driver mutation identification. bioRxiv.

